# Outpatient inguinal hernia repair in Spain: a population-based study of 1,163,039 patients—clinical and socioeconomic factors associated with the choice of day surgery

**DOI:** 10.1007/s13304-022-01407-1

**Published:** 2022-10-26

**Authors:** Salvador Guillaumes, Nils Jimmy Hidalgo, Irene Bachero, Montserrat Juvany

**Affiliations:** 1 Department of Gastrointestinal Surgery, Hospital Clinic de Barcelona (Seu Plató), C/Plató 21, 08006 Barcelona, Spain; 2 Department of Surgery, Hospital Universitari de Granollers, Granollers, Spain

**Keywords:** Inguinal hernia, Outpatient surgery, Day surgery, Trend analysis, Epidemiology

## Abstract

Reducing inpatient admissions and health care costs is a central aspiration of worldwide health systems. This study aimed to evaluate trends in outpatient surgery in inguinal hernia repair (IHR) and factors related to the outpatient setting in Spain. A retrospective cohort study (Record-Strobe compliant) of 1,163,039 patients who underwent IHR from January 2004 to December 2019 was conducted. Data were extracted from the public clinical administrative database CMBD (“Conjunto Mínimo Básico de Datos”). The primary outcome was the outpatient surgery rate. Univariate and multivariable analyses were performed to identify clinical and socioeconomic factors related to the outpatient setting. The overall proportion of outpatient repairs was 30.7% in 2004 and 54.2% in 2019 (*p* < 0.001). Treatment in a public hospital was the most remarkable factor associated with the likelihood of receiving an outpatient procedure (OR 3.408; *p* < 0.001). There were also significant differences favouring outpatient procedures for patients with public insurance (OR 2.351; *p* < 0.001), unilateral hernia (OR 2.903; *p* < 0.001), primary hernia (OR 1.937; *p* < 0.0005), age < 65 years (OR 1.747; *p* < 0.001) and open surgery (OR 1.610; *p* < 0.001). Only 9% of patients who pay for their intervention privately or 15% of those covered by private insurance were treated as outpatients. Spain has significantly increased the rate of outpatient IHR over the last 16 years. However, the figures obtained still leave a significant margin for improvement. Important questions about the acceptance of outpatient settings remain to be answered.

**Graphical Abstract:**

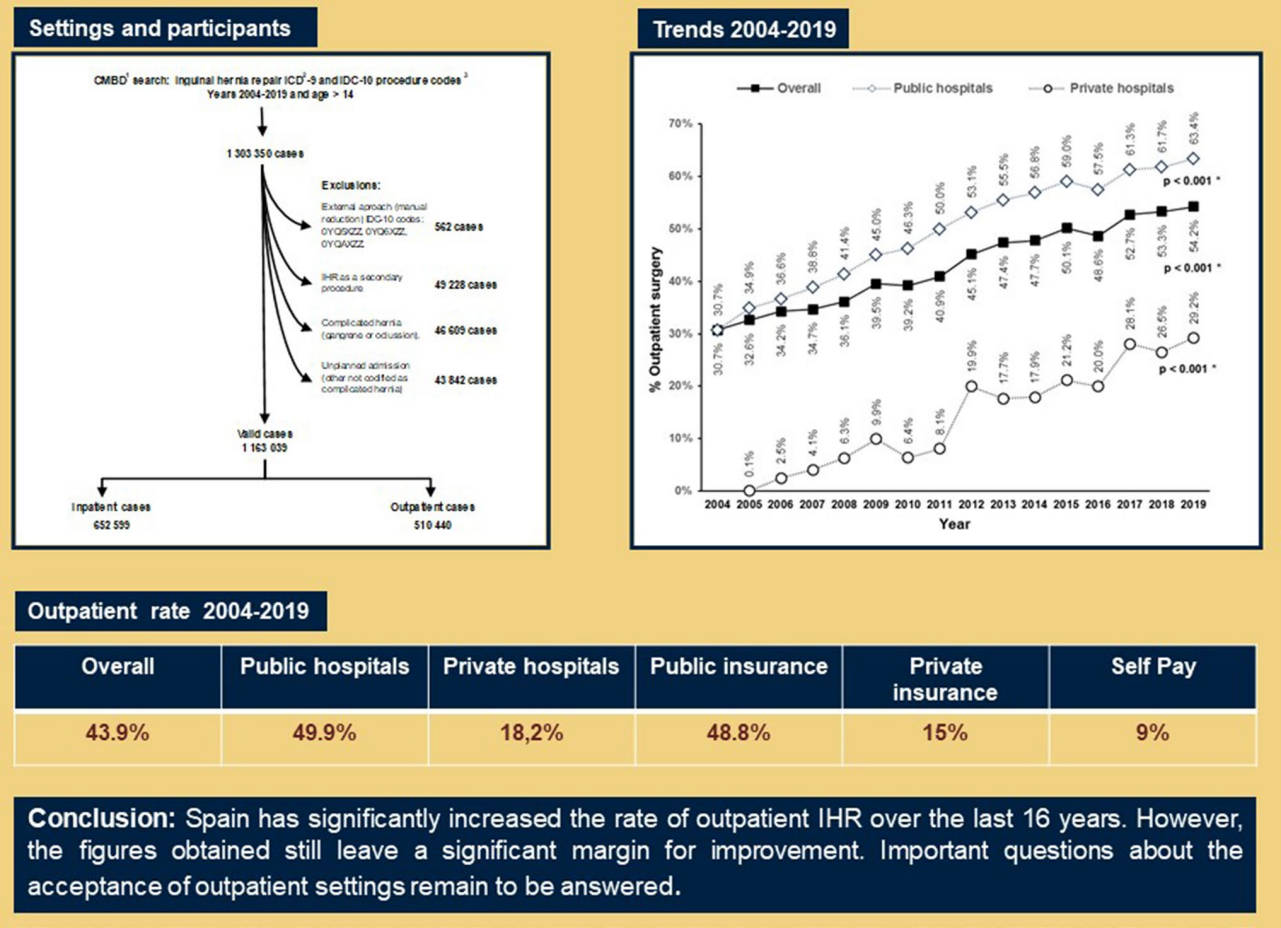

Outpatient inguinal hernia repair in Spain. A population-based study of 1,163,039 patients: clinical and socioeconomic factors associated with the choice of day surgery.

**Supplementary Information:**

The online version contains supplementary material available at 10.1007/s13304-022-01407-1.

## Introduction

Inguinal hernia repair (IHR) is one of the most commonly performed surgical procedures worldwide; it is estimated that over 20 million inguinal hernias are repaired each year [1], with over 90,000 performed in Spain [2]. The international guidelines for groin hernia management recommend outpatient procedures, including when using the laparoscopic technique [1]. A high level of consensus supports this recommendation, provided adequate aftercare is organized [3].

Improvements and innovations in surgical procedures and anaesthesia techniques have enabled a shift to outpatient settings [4]. Outpatient surgeries are generally less expensive because they require fewer personnel, fewer resources, and less infrastructure [5]. Much effort has been made to reduce healthcare spending by promoting outpatient surgeries [4–6]. In addition to the beneficial effects on costs and sustainability of the health system, outpatient surgery has shown beneficial effects on the quality of life of patients who recover at home without having negative effects on the number of complications or the recurrence percentages [5, 7–9].

Norway, Sweden, Denmark, the Netherlands, and the United States (US) were early adopters of day surgery, where the rates of outpatient inguinal hernia repair reached 75%. In contrast, Germany, Austria, and Eastern European countries were low adopters [10]. In Spain, the National Health Service indicators show an overall 57% rate of outpatient IHR in public hospitals [11].

Spain, like other countries, has a mandatory national hospital patient discharge registry, the National Minimum Basic Dataset (CMBD, Spanish acronym for Conjunto Minimo Básico de Datos), which is collected in hospitals and curated and published annually by the Ministry of Health. This clinical and administrative database, to which all Spanish hospitals are required to submit validated, and reliable data [12, 13], has previously been utilized as a research tool in inguinal hernias [2, 14] and other diseases [15]. Such types of databases have also been used in large inguinal hernia series in the US, Germany, and other countries [16–20].

The primary objective of this study was to determine the proportion of IHR carried out in the outpatient setting, in both public and private hospitals, and to analyse the trends over 16 years using data from the CMBD national database. The second objective was to identify the clinical, demographic and socioeconomic factors associated with the choice of an outpatient procedure.

## Methods

### Study design

We conducted a retrospective cohort analysis (Record-Strobe compliant) based on official data requested from the Ministry of Health. Data came from the Discharges Record on Hospitalization and Specialized Out-Patient Care recorded in the Spanish CMBD [12, 13]. The Spanish CMBD includes anonymized personal and clinical information from patients discharged in all public and private hospitals in Spain. Reporting the data to the Ministry is mandatory and is usually linked to the billing and financing of public hospitals. The database collects 100% of hospital discharges from the public healthcare system and, since 2012, it also includes about 90% of discharges from private centres.

This data set currently includes 31 clinical and administrative variables that collect information about patients’ characteristics (age, gender and place of residence), primary and secondary diagnoses, which include comorbidities, procedures performed, hospital stay, and perioperative mortality. From 2004 to 2015, the diagnoses and procedures in the CMBD were coded using the International Classification of Diseases, Ninth Revision, Clinical Modification. (ICD-9-CM). The International Classification of Diseases, Tenth Revision, Clinical Modification (ICD-10-CM) was used to code data from 2016 to 2019 (the most recent year available).

### Study population

**Fig. 1:**
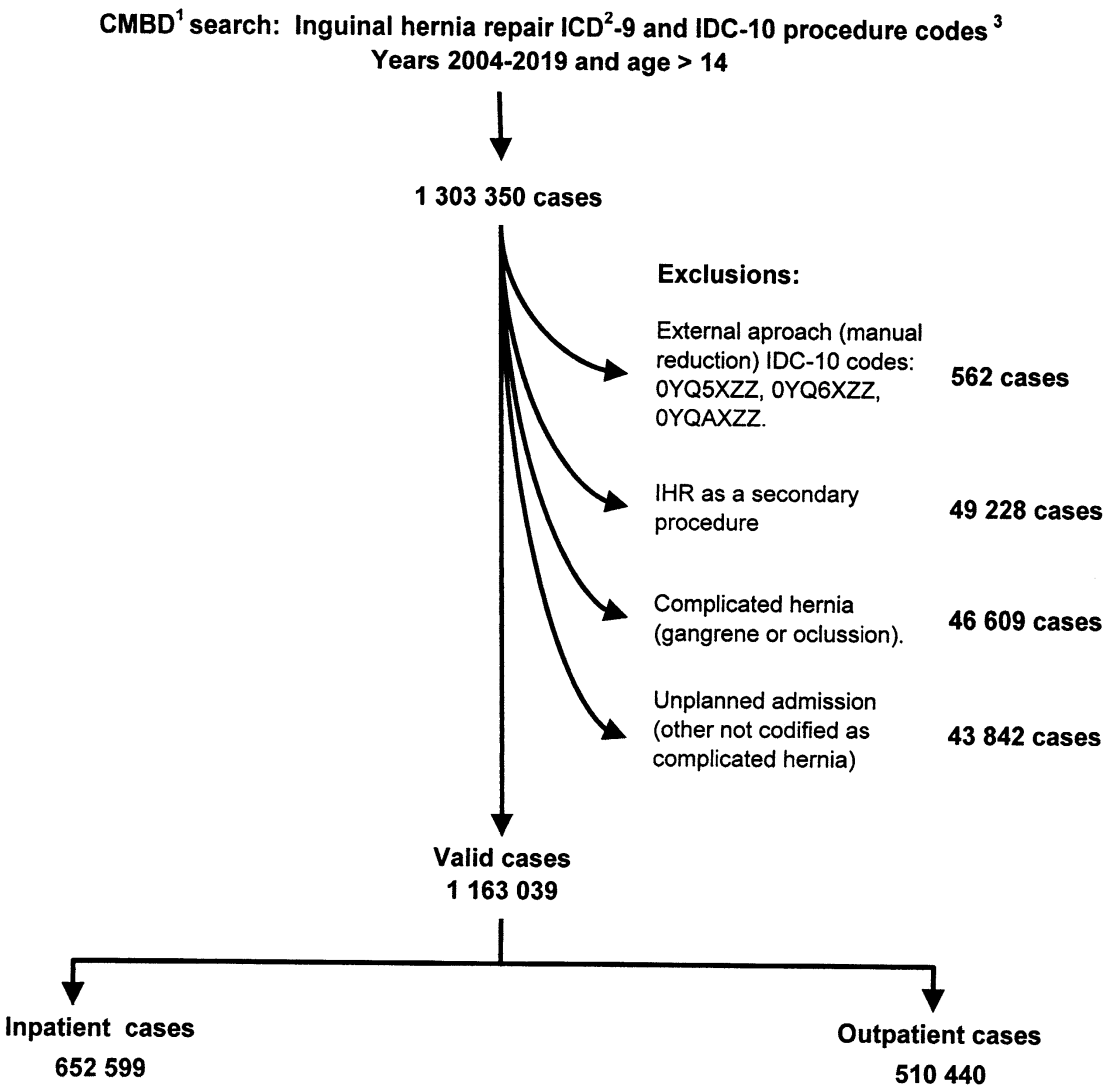
Flowchart illustrating the selection of inguinal hernia repairs, exclusions and approach classification ([1]. *CMBD* health ministry database; [2] *ICD* international classification of diseases, [3] the ICD codes used in search are detailed in supplementary Document_1)

All patients over 14 years who underwent an IHR between January 1st, 2004, and December 31st, 2019, were included. The flowchart (Fig. 1) depicts the procedure used to identify patients as well as the exclusion criteria that have been established. ICD-9 and ICD-10 surgical procedure codes (Supplementary Document_1) were used to identify patients The procedure codes allow the classification of unilateral and bilateral cases, and open or laparoscopic approaches (the latter aspect only from 2010 and with irregular implantation until the application of the ICD-10 in 2016). ICD-9 and ICD-10 diagnostic codes (also included in Supplementary Document_1) allow the identification of primary or recurrent hernias and complicated hernias with bowel obstruction or gangrene.

### Data collection

Administrative covariates included: scheduled or emergency procedures, standard hospitalization or outpatient surgery, hospital ownership (public or private), and patient financing options (public insurance, private insurance, self-pay or other funds). Other collected variables were hospital stay and mortality (patients discharged dead). Patient-related covariates included sex, age, unilateral or bilateral repair and secondary diagnoses as comorbidities (chronic obstructive pulmonary disease, arterial hypertension, diabetes, dyslipidaemia, obesity and tobacco consumption/smoking) or postoperative complications (wound infection, haemorrhage/haematoma and acute urinary retention). All secondary diagnoses (comorbidities and complications) were identified using ICD-9 and ICD-D-10 codes (included in supplementary Document_1). The complications are only registered in the CMBD database if they are identified before the patient is discharged. As a result, the usual complication rates for a 30-day postoperative period are unavailable. We used the Ministry of Health population database [21] to obtain national and autonomous community population data and the proportion of people over the age of 65. The data used to calculate population density (population per Km^2^) and gross domestic product (GDP) per capita for each autonomous community were obtained from the web server of the National Statistics Institute of Spain [22].

### Statistical analysis

The SAMPL guidelines for basic statistical reporting [23] were followed. A descriptive analysis was performed to explore differences between outpatient (day surgery) and inpatient surgery cases. The categorical variables were presented as frequencies, percentages and the odds ratio (OR) estimate, using the Chi-squared (χ2) test to compare groups. Quantitative results were expressed as the mean and standard deviation (SD) and the data were compared using the Student’s *t*-test for independent samples. We evaluated the trend over time of outpatient surgery cases using a one-tailed Cochran–Armitage test [24]. A multivariate logistic regression model was used to estimate the OR and evaluate the factors associated with outpatient surgery. The independent predictor variables included in the model were: hospital owner (public), insurance (public), laterality (unilateral), recurrent hernia (no), sex (female), age (< 65years), surgical approach (open) and associated comorbidities (chronic obstructive pulmonary disease, hypertension, diabetes, obesity or smoking). Pearson correlation coefficients were used to investigate the relationship between the outpatient rate in autonomous communities and sociodemographic variables such as population density, GDP per capita or population ageing. Less than 1% of cases have missing data, which has been eliminated pairwise. Statistical significance was established when *p* values were less than 0.05. All statistical analyses were performed using the IBM SPSS^®^ Statistics for Windows^®^, version 20.0 (IBM Corp, Chicago, IL, USA).

### Ethical considerations

All data analysed are anonymous and were extracted from a public computerized database upon request [13]. The database is managed by the Spanish Ministry of Health. Therefore, this study did not need the approval of an Ethics Committee for Medical Research. The informed consent of the patients was previously obtained by every hospital before the surgical procedure.

## Results

A total of 1,163,039 patients met the final inclusion criteria for the study. With a mean (SD) age of 58.83 (15.44) years, there were 1,043,077 male patients (89.7%) and 142,807 female patients (12.3%). Patients were divided into two groups based on whether they had outpatient or inpatient surgery: 510,440 (43.89%) had outpatient surgery and 652,599 (56.11%) had inpatient surgery.

### Trends in the rate of outpatient surgery

**Fig. 2:**
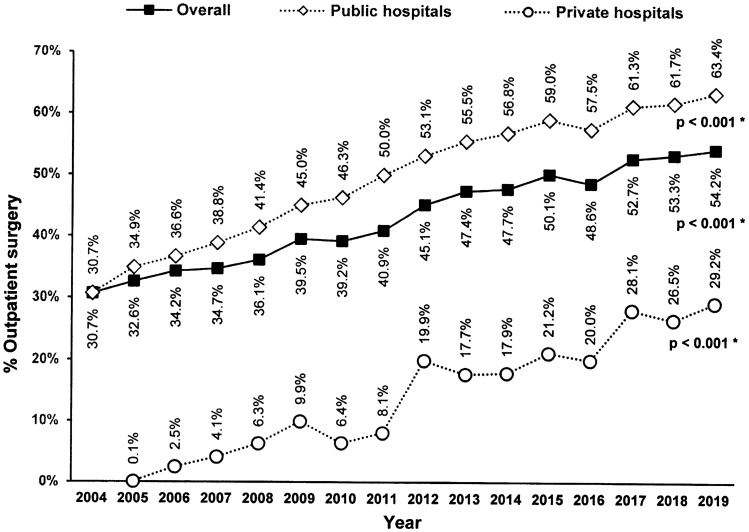
Rate of inguinal hernia repair (*IHR*) performed on an outpatient basis in Spain, from 1 January 2004 to 31 December 2019, stratified by a public or private hospital. ([*] Cochran–Armitage trend test)

Figure 2 shows the trend over the years in the rate of outpatient settings. The overall proportion of outpatient repairs was 30.7% in 2004 and 54.2% in 2019 (*p* < 0.001 Cochran–Armitage trend test). In public hospitals, the proportion was 30.7% in 2004 and 63.4% in 2019 (*p* < 0.001), while in private hospitals, it was 0.1% in 2005 and 29.2% in 2019 (*p* < 0.001).

### Characteristics of the cohort by outpatient or inpatient surgery setting

Table 1 shows the demographic, clinical and socioeconomic differences of the cohort by outpatient or inpatient surgery setting. The age range distribution between both groups was statistically different, with a higher proportion of younger patients (age ranges 15–44 and 45–64 years) in the outpatient group (Mean (SD) age of 55.62 (15.01) vs. 61.34 (15.3) years; *p* < 0.001, *t*-test). The gender differences were minimal. In the outpatient IHR group, there were more unilateral cases (95.2 vs. 87.8%, OR 2.712; *p* < 0.001), more primary cases (95.4 vs. 91.3%, OR 1.918; *p* < 0.001) and fewer comorbidities.

**Table 1:** Patient clinical and socioeconomic characteristics by inpatient or outpatient cases

	Overall	Inpatient	Outpatient	OR^a^	CI^b^ 95%		p*
	*n*	*n* (%)	*n* (%)				
All cases	1,163,039	652,599 (56.11%)	510,440 (43.89%)				
Age < = 65 years	701,918	347,824 (53.3%)	354,094 (69.4%)	1.985	1.969	2.000	< 0.001
Age groups							
15–44 years	222,038	100,682 (15.4%)	121,356 (23.8%)	1.710	1.694	1.726	< 0.001
45–64 years	479,880	247,142 (37.9%)	232,738 (45.6%)	1.375	1.365	1.385	< 0.001
65–74 years	296,197	162,026 (24.8%)	102,767 (20.1%)	0.763	0.757	0.770	< 0.001
> 74 years	196,328	142,749 (21.9%)	53,579 (10.5%)	0.419	0.414	0.423	< 0.001
Gender							
Male	1,043,077	587,479 (90.0%)	455,598 (89.3%)	0.921	0.910	0.932	< 0.001
Female	142,807	65,020 (10.0%)	54,772 (10.7%)	1.086	1.073	1.099	< 0.001
Laterality							
Unilateral repair	1,058,384	572,862 (87.8%)	485,522 (95.2%)	2.712	2.672	2.752	< 0.001
Bilateral repair	104,655	79,737 (12.2%)	24.918 (4.9%)	0.369	0.363	0.374	< 0.001
Recurrence							
No (primary hernia)	1,082,839	596,082 (91.3%)	486,757 (95.4%)	1.949	1.918	1.979	< 0.001
Recurrent hernia	80,200	56,517 (8.7%)	23,683 (4.6%)	0.513	0.505	0.521	< 0.001
Comorbidities							
Hypertension	209,924	156,055 (23.9%)	53,869 (10.6%)	0.375	0.371	0.379	< 0.001
Dyslipidaemia	111,224	78,825 (12.1%)	32,399 (6.3%)	0.493	0.487	0.500	< 0.001
Diabetes	64,787	48,906 (7.5%)	15,881 (3.1%)	0.396	0.389	0.404	< 0.001
COPD^c^	47,299	37,122 (5.7%)	10,177 (2.0%)	0.337	0.330	0.345	< 0.001
Obesity	15,733	10,173 (1.6%)	5,560 (1.1%)	0.695	0.673	0.719	< 0.001
Tobacco use	69,427	42,708 (6.5%)	26,719 (5.2%)	0.789	0.776	0.801	< 0.001
Surgical approach							
Open	1,132,465	633,882 (97.1%)	498,583 (97.7%)	1.241	1..213	1.271	< 0.001
Laparoscopic	30,574	18,717 (2.9%)	11,857 (2.3%)	0.805	0.787	0.824	< 0.001
Insurance type							
Public	977,943	500,999 (76.8%)	476,944 (93.4%)	4.309	4.255	4.363	< 0.001
No insurance/self-pay	72,461	65.418 (10.0%)	7,043 (1.4%)	0.126	0.122	0.129	< 0.001
Private insurance	46,001	38,704 (5.9%)	7,297 (1.4%)	0.230	0.224	0.236	< 0.001
Other funds	66,634	47,478 (7.3%)	19,156 (3.8%)	0.497	0.488	0.506	< 0.001
Hospital ownership							
Public	942,889	472,409 (72.4%)	470,480 (92.2%)	4.491	4.439	4.543	< 0.001
Private	220,150	180,190 (27.6%)	39,960 (7.8%)	0.223	0.220	0.225	< 0.001

*Chi-square test

^a^*OR* odds ratio

^b^*CI* confidence interval

^c^*COPD* chronic obstructive pulmonary disease

Outpatient IHR patients were more likely to be treated in public hospitals (92.2 vs. 72.4% in private hospitals; *p* < 0.001). Patients undergoing surgery in private hospitals (220,150) account for 18.9% of the total, with 82% being inpatients and only 18% being outpatients. The analysis also found that 93.4% of outpatient surgery patients had financial coverage from public insurance, compared to only 76.8% of inpatient surgery patients (*p* < 0.001). Only 9% of patients who pay for their intervention privately, or 15% of those with private insurance, were treated as outpatients.

Analysis of the entire series showed that the percentage of patients who underwent a laparoscopic procedure was only 2.6%. In a separate analysis of the year 2019 cases, the laparoscopic IHR reached 8.5%, and the outpatient surgery rate was significantly higher in open surgery patients (60.3 vs. 39.7%; *p* < 0.001).

### Multivariable analysis

The multivariate logistic regression analysis (Table 2) revealed that surgery performed in a public hospital was the most remarkable factor associated with the likelihood of receiving outpatient surgery (OR 3.408; *p* < 0.001). There were also significant differences in multivariate analysis favouring outpatient surgery for patients having public insurance (OR 2.351; *p* < 0.001), unilateral operation (OR 2.903; *p* < 0.001), primary hernia (OR 1.937; *p* < 0.001), age younger than 65 years (OR 1.747; *p* < 0.001) and open surgery (OR 1.610; *p* < 0.001). Patients with comorbidities (such as chronic obstructive pulmonary disease, diabetes, or obesity) were less likely to undergo outpatient surgery.

**Table 2:** Multivariate analysis of clinical and socioeconomic factors associated with outpatient surgery in inguinal hernia repair

	OR^a^	95% CI^b^ for OR		p*
		Lower	Upper	
Public hospital	3.408	3.358	3.458	0.001
Unilateral repair	2.903	2.858	2.948	0.001
Public insurance	2.351	2.313	2.388	0.001
Primary hernia	1.937	1.905	1.970	0.001
Age < 65 years	1.747	1.732	1.762	0.001
Open surgery	1.610	1.568	1.654	0.001
Sex female	1.056	1.042	1.070	0.001
Obesity	0.854	0.824	0.885	0.001
Tobacco use	0.807	0.793	0.821	0.001
Diabetes	0.577	0.565	0.588	0.001
Hypertension	0.449	0.444	0.455	0.001
COPD^c^	0.411	0.402	0.421	0.001

*logistic regression

^a^*OR* odds ratio

^b^*CI* confidence interval

^c^*COPD* chronic obstructive pulmonary disease

### Postoperative outcomes

Immediate postoperative complications were significantly lower in the outpatient group, with a lower rate of haemorrhage/haematoma (0.025 vs. 1.17%, OR 47.0; *p* < 0.001), retention of urine (0.026 vs. 0.31%, OR 12.3; *p* < 0.001) and wound infection (0.062 vs. 0.0018%, OR 35.1; *p* < 0.001). Overall mortality (discharged dead) in this series was 0.017% (198/1,163,039 patients), 0.0043% in outpatients (22/510,440 patients), and 0.027% in inpatients (OR 6.3; *p* < 0.001).

### Geographic variability in the outpatient rates

A separate analysis was done, only with the year 2019 cases, to demonstrate variability across the country (Table 3). The outpatient rate in Spain’s autonomous communities, which are the country’s first political and administrative divisions, varies from 3 to 81.8%. Variations in outpatient rates in each autonomous community do not correlate with population density, GDP per capita or population aging.

**Table 3:** Outpatient inguinal hernia repair percentages in Autonomous Communities (AACC) in 2019

AACC	Inguinal hernia cases (year 2019)			AACC sociodemographic characteristics			
	Total	Outpatient			GDP^#^	Ageing^€^	
	*n*	*n*	%	Hab/Km^2^	Per capita	≥ 65 years	
Andalusia	15.258	9.788	**64.1**	96.4	19.530		13.4%
Aragón	2.239	427	**19.1**	27.8	28.759		17.3%
Asturias	1.586	755	**47.6**	96.2	23.240		25.9%
Balearic Islands	859	554	**64.5**	240.0	28.522		15.9%
Canary Islands	3.620	1.638	**45.2**	298.1	21.387		16.4%
Cantabria	988	674	**68.2**	109.4	24.350		22.1%
Castille and León	5.386	1.132	**21.0**	25.5	24.910		25.4%
Castille-La Mancha	3.662	2.350	**64.2**	25.7	20.841		19.0%
Catalonia	13.881	7.382	**53.2**	237.0	31.209		19.2%
Valencian Com	10.451	6.980	**66.8%**	215.0	23.083		19.6%
Extremadura	1.854	1.189	**64.1%**	25.5	19.304		20.7%
Galicia	4.960	2.585	**52.1**	91.3	23.842		20.0%
Com. of Madrid	13.081	7.300	**55.8**	832.9	36.049		17.9%
Región of Murcia	2.829	1.544	**54.6**	132.1	21.596		15.8%
Navarre	1.510	860	**57.0**	62.8	32.030		19.9%
Basque country	5.447	2.305	**42.3**	301.6	33.938		17.8%
La Rioja	548	331	**60.4**	62.3	28.128		17.7%
Ceuta^¥^	77	63	**81.8**	4223.5	20.960		10.2%
Melilla^¥^	66	2	**3.0**	7033.3	19.224		9.7%

Correlation coefficients			
	Hab/Km^2^	GPD	Ageing
Outpatient %			
Pearson correlation	0.102	−0 228	−0 204
Sig. (2-tailed)	0.697	0.379	0.432
N	17	17	17

^#^GDP 2019 gross domestic product

^€^2019% of population ≥ 65 years

^ ¥^*a*utonomous cities in Nord Africa with a small number of cases and high population density were excluded from the analysis

## Discussion

### Rate of outpatient surgery. What proportion of patients could be expected to be eligible for an outpatient procedure?

The main observations of our study are the significant increase in outpatient IHR over the study period (30 to 54.2%) and the predominance of outpatient cases in public hospitals, with a much lower presence in private centres or when patients with private insurance or private payment are taken into account.

It is difficult to establish what proportion of patients could be expected to be eligible for an outpatient procedure. The percentage of patients who, for social or medical reasons, are not eligible for outpatient IHR has been classically established within limits ranging from 3 to 17% [25, 26]. In another study, Solodkyy [27] found that of 1000 cases of laparoscopic IHR in an outpatient unit in the United Kingdom, only 822 (82%) were finally true day surgery cases; 8.5% were considered unsuitable and were rescheduled for inpatient surgery, and 10.2% stayed overnight unexpectedly.

The guidelines from the Hernia Surge Group [1] recommend outpatient surgery in the majority of patients, including laparoscopic repair of simple inguinal hernias, and also in selected older ASA IIIa patients, provided adequate aftercare is organized. Exclusion criteria include complex inguinal hernias, strangulated and acutely incarcerated cases, patients with anticoagulant treatment, nonagenarians, and cases with intraoperative bleeding or other complications. They do not provide an objective figure, although they cite the countries with the greatest outpatient surgery implantation, such as Sweden (75%) or the Northern Italian Veneto region (87%). According to a report from the Royal Australasian College of Surgeons [28], “the target rate for hospitals should be between 70 and 80% of patients as same-day cases.” Similarly, the clinical guidelines of the Association of Surgeons of Great Britain and Ireland and the British Hernia Society establish an outpatient surgery rate of more than 70% as a quality indicator [29].

We may consider achieving 70% of outpatient cases a reasonable objective in our series. Therefore, the overall rate found (54.2% in 2019) leaves a significant margin for improvement.

### Factors related to the outpatient setting

Other study findings include lower rates of ambulatory surgery in patients over the age of 65, patients with comorbidities, and patients with bilateral and recurrent hernias, all of which are common in other countries and series.[17, 30, 31]. Data concerning other factors, such as high body mass index, intervention duration, or patient slot in the operating list [27], are not available in our clinical administrative database. More interesting topics to discuss could include the economic implications of outpatient surgery, the role of care financing, regional differences in regulations or incentives, and the impact of the rise in laparoscopic IHR.

The primary motivation for moving surgery from an inpatient to an outpatient setting is cost savings while maintaining the same quality and safety of care [4]. In Spain, the hospital costs of outpatient surgery are between 25 and 68% lower than those of inpatient surgery for the same procedure [32]. Furthermore, outpatient surgery avoids hospital stays, which allows for treating a greater number of patients and reducing waiting lists. Economic incentives to the patient, hospital and/or surgeon may promote or discourage the practice of day surgery depending on the arrangement [33].

In the US, the significantly lower cost of outpatient surgical procedures and, therefore, the cost to be assumed by the patient in co-payment are determinant factors that favour, in some cases, the choice of outpatient surgery [34]. In the Italian Veneto region, where there is a high outpatient surgery rate, the Regional Authority has set a specific regional target of 13% inpatient IHR: when this target is exceeded, the price assumed by the government for ordinary admission for hernia repair drops in order to encourage a limitation of the use of inpatient care [6]. The low rate of outpatient surgery found in our review in some autonomous communities may be related to differences in incentives in these communities. In Switzerland, legislative differences between cantons induce similar variability [4, 5].

On the contrary, according to a recent study that included cases from “Herniamed,” an internet-based hernia registry in which hospitals and independent surgeons in Germany, Austria and Switzerland voluntarily enter their cases, the percentage of outpatient IHR has decreased in recent years; the proportion of outpatient repairs was 20.2% in 2013 and 14.3% in 2019 [30]. This decrease may be related in part to the expansion of laparoscopic repair. The proportion of laparoscopic repairs among the inpatient cases was 71.9% in 2019 and only 34.3% for outpatient cases [30]. In Germany, where much of the financing is covered by private insurance, several studies have shown that the German reimbursement system does not adequately cover the costs for outpatient laparoscopic repairs and there is no incentive for outpatient hernia surgeries [30]. In contrast, reimbursement covers the cost of laparoscopic surgery for inpatient hernia repair [30]. In Spain, where laparoscopic surgery is still a minority [14], we found a significantly lower percentage of outpatient surgeries, and only 38.8% of laparoscopic repairs were outpatient cases. We hope that the future development of laparoscopic surgery, a technique that favours outpatient surgery [27], will be an incentive rather than an impediment to expanding the number of outpatient surgeries.

In our results, it is striking how only 9% of patients who privately paid for their intervention or 15% of those who were financed by private insurance were finally operated on an outpatient basis. This result might depend on an individual’s preference for an overnight stay (82% of private IHR patients in our series stay overnight) or alternatively, on economic or logistical reasons of the service provider. In older studies, a significant proportion of patients preferred an overnight stay [35, 36]. More recent studies found high satisfaction levels in patients who operated on an outpatient basis [37]. Although patient satisfaction with day surgery is high, information provision and pain management at home remain the greatest challenges [38, 39] and they may determine low acceptance of day surgery.

### Identifying questions about the acceptance of outpatient settings and areas of improvement

The low acceptance of ambulatory surgery in the field of surgery financed by self-pay or private insurance, as well as the ongoing room for improvement in public medicine, raises some questions about the acceptance of ambulatory surgery by patients, or even by professionals, and highlights the need for strategies that allow ambulatory surgery to progress.

Adequate promotion and education are mechanisms to increase patient acceptability and satisfaction. People will have more confidence in ambulatory surgical care processes if they fully understand that they will get all the necessary preoperative and postoperative support and information, as well as the most rigorous pain control. A further promotion method is to establish incentives for professionals and service providers.

### Strengths and limitations of the study

The most important strengths of our findings are the high degree of reliability provided by data collected from the same source for 16 years and the high statistical power provided by the large sample size. It is also a strength that the variable analysed as the main endpoint (inpatient or outpatient surgery) is a key administrative piece of data in the management and billing of hospitals and therefore is a variable with very few possibilities of mistakes.

Our study had several limitations. First, the CMBD database includes a limited number of variables without the option to link to other databases. Although it contained a large number of patients, the level of clinical detail was limited. We had no data on operative outcomes on the usual 30-day follow-up or data about hernia recurrence, and there is a clear selection bias in the analysis of immediate postoperative complications due to the selection of the outpatient cases. Furthermore, like all administrative databases, our database contains only the discharge data included in the discharge report, which may be incomplete. Later, when the administrative staff codifies the report, this may result in mistakes. Additionally, it must be kept in mind when interpreting the results that, given the large cohort size, small differences between the groups may be statistically significant and, therefore, this statistical significance may not be relevant. Likewise, we must indicate the limitations of the correlation coefficients used to suggest the non-association of the percentages of outpatient surgery with the sociodemographic variables of the autonomous communities.

Despite these limitations, the CMBD database is an extraordinarily powerful source of information. Its usefulness has been previously demonstrated in observational and epidemiological studies carried out in Spain [2, 14, 15].

## Conclusions

In the last 16 years, Spain has substantially increased the rate of outpatient IHR. However, the figures achieved still leave a significant margin for improvement, and important questions about the acceptance of outpatient settings remain to be answered. Efforts will be required by the health administration and the medical and nursing staff to increase acceptance of outpatient surgery and to achieve the expected objectives.

## Supplementary Information

Below is the link to the electronic supplementary material.

Supplementary file1 (DOCX 19 KB)

## Data Availability

All the data analysed came from a public database managed by Spain’s Ministry of Health. Researchers from public and private institutions can request data by filling out a questionnaire on the Ministry's website. This questionnaire requires a signed confidentiality agreement. According to the confidentiality agreement, researchers cannot share their data with other researchers; instead, they must request the data directly from the Ministry. Alternatively, a large part of the data can be consulted openly through the interactive consultation system of the Spanish National Health System. https://pestadistico.inteligenciadegestion.sanidad.gob.es/publicoSNS/N/rae-cmbd/rae-cmbd
