# Supplementary material for: Outpatient inguinal hernia repair in Spain: a population-based study of 1,163,039 patients—clinical and socioeconomic factors associated with the choice of day surgery
Source: Updates Surg. 2022 Oct 26;75(1):65–75. doi: 10.1007/s13304-022-01407-1 (PMC9834115; doi:10.1007/s13304-022-01407-1)
Supplement: Supplementary file 1 — Supplementary file1 (DOCX 19 KB) [file 13304_2022_1407_MOESM1_ESM.docx]

**Outpatient inguinal hernia repair in Spain. A population based study of 1,163,039 patients: clinical and socioeconomic factors associated with the choice of day surgery.**

**Supplementary Document_1.**

**International Classification of Diseases (ICD) codes** **used to identify patients and clinical variables.**

Data from 2004 to 2015 were coded using, Ninth Revision, Clinical Modification (ICD-9-CM). Data from 2016 to 2019 were coded using the International Classification of Diseases, Tenth Revision, Clinical Modification (ICD-10-CM).

**ICD-9 Surgical procedure codes** **used to identify the patients**

53.0 Other Unilateral Repair Of Inguinal Hernia

53.00 Unilateral repair of inguinal hernia, not otherwise specified

53.01 Other and open repair of direct inguinal hernia

53.02 Other and open repair of indirect inguinal hernia

53.03 Other and open repair of direct inguinal hernia with graft or prosthesis

53.04 Other and open repair of indirect inguinal hernia with graft or prosthesis

53.05 Repair of inguinal hernia with graft or prosthesis, not otherwise specified

53.1 Other Bilateral Repair of Inguinal hernia

53.10 Bilateral repair of inguinal hernia, not otherwise specified

53.11 Other and open bilateral repair of direct inguinal hernia

53.12 Other and open bilateral repair of indirect inguinal hernia

53.13 Other and open bilateral repair of inguinal hernia, one direct and one indirect

53.14 Other and open bilateral repair of direct inguinal hernia with graft or prosthesis

53.15 Other and open bilateral repair of indirect inguinal hernia with graft or prosthesis

53.16 Other and open bilateral repair of inguinal hernia, one direct and one indirect, with graft or prosthesis

53.17 Bilateral inguinal hernia repair with graft or prosthesis, not otherwise specified

17.1 Laparoscopic Unilateral Repair Of Inguinal Hernia

17.11 Laparoscopic repair of direct inguinal hernia with graft or prosthesis

17.12 Laparoscopic repair of indirect inguinal hernia with graft or prosthesis

17.13 Laparoscopic repair of inguinal hernia with graft or prosthesis, not otherwise specified

17.2 Laparoscopic Bilateral Repair Of Inguinal Hernia

17.21 Laparoscopic bilateral repair of direct inguinal hernia with graft or prosthesis

17.22 Laparoscopic bilateral repair of indirect inguinal hernia with graft or prosthesis

17.23 Laparoscopic bilateral repair of inguinal hernia, one direct and one indirect, with graft or prosthesis

17.24 Laparoscopic bilateral repair of inguinal hernia with graft or prosthesis, not otherwise specified

**ICD-10 Surgical procedure codes used to identify the patients**

**Medical and Surgical. Anatomical Regions Lower Extremities. Repair. Inguinal Region, Right**

0YQ50ZZ Repair Right Inguinal Region. No device. Open Approach

0YQ53ZZ Repair Right Inguinal Region. No device. Percutaneous Approach

0YQ54ZZ Repair Right Inguinal Region. No device. Percutaneous Endoscopic Approach

0YQ5XZZ Repair Right Inguinal Region. No device. External Approach

**Medical and Surgical. Anatomical Regions Lower Extremities. Repair** **Inguinal Region, Left**

0YQ60ZZ Repair Left Inguinal Region. No device. Open Approach

0YQ63ZZ Repair Left Inguinal Region, No device. Percutaneous Approach

0YQ64ZZ Repair Left Inguinal Region. No device. Percutaneous Endoscopic Approach

0YQ6XZZ Repair Left Inguinal Region. No device. External Approach

**Medical and Surgical. Anatomical Regions Lower Extremities. Repair Inguinal Region, Bilateral**

0YQA0ZZ Repair Bilateral Inguinal Region. No device. Open Approach

0YQA3ZZ Repair Bilateral Inguinal Region. No device. Percutaneous Approach

0YQA4ZZ Repair Bilateral Inguinal Region. No device. Percutaneous Endoscopic Approach

0YQAXZZ Repair Bilateral Inguinal Region. No device. External Approach

**Medical and Surgical. Anatomical Regions Lower Extremities. Supplement. Inguinal Region, Right**

0YU507Z Supplement Right Inguinal Region with Autologous Tissue Substitute, Open Approach

0YU50JZ Supplement Right Inguinal Region with Synthetic Substitute, Open Approach

0YU50KZ Supplement Right Inguinal Region with Nonautologous Tissue Substitute, Open Approach

0YU547Z Supplement Right Inguinal Region with Autologous Tissue Substitute, Percutaneous Endoscopic Approach

0YU54JZ Supplement Right Inguinal Region with Synthetic Substitute, Percutaneous Endoscopic Approach

0YU54KZ Supplement Right Inguinal Region with Nonautologous Tissue Substitute, Percutaneous Endoscopic Approach

**Medical and Surgical. Anatomical Regions Lower Extremities. Supplement. Inguinal Region, Left**

0YU607Z Supplement Left Inguinal Region with Autologous Tissue Substitute, Open Approach

0YU60JZ Supplement Left Inguinal Region with Synthetic Substitute, Open Approach

0YU60KZ Supplement Left Inguinal Region with Nonautologous Tissue Substitute, Open Approach

0YU647Z Supplement Left Inguinal Region with Autologous Tissue Substitute, Percutaneous Endoscopic Approach

0YU64JZ Supplement Left Inguinal Region with Synthetic Substitute, Percutaneous Endoscopic Approach

0YU64KZ Supplement Left Inguinal Region with Nonautologous Tissue Substitute, Percutaneous Endoscopic Approach

**Medical and Surgical. Anatomical Regions Lower Extremities. Supplement. Inguinal Region, Bilateral**

0YUA07Z Supplement Bilateral Inguinal Region with Autologous Tissue Substitute, Open Approach

0YUA0JZ Supplement Bilateral Inguinal Region with Synthetic Substitute, Open Approach

0YUA0KZ Supplement Bilateral Inguinal Region with Nonautologous Tissue Substitute, Open Approach

0YUA47Z Supplement Bilateral Inguinal Region with Autologous Tissue Substitute, Percutaneous Endoscopic Approach

0YUA4JZ Supplement Bilateral Inguinal Region with Synthetic Substitute, Percutaneous Endoscopic Approach

0YUA4KZ Supplement Bilateral Inguinal Region with Nonautologous Tissue Substitute, Percutaneous Endoscopic Approach

**ICD 9 Diagnostic codes used to identify patients with primary or recurrent inguinal hernia and, with or without, gangrene or occlusion at presentation.**

550.0 Inguinal hernia with gangrene

550.00 Inguinal hernia, with gangrene, unilateral or unspecified (not specified as recurrent)

550.01 Inguinal hernia, with gangrene, unilateral or unspecified, recurrent

550.02 Inguinal hernia, with gangrene, bilateral (not specified as recurrent)

550.03 Inguinal hernia, with gangrene, bilateral, recurrent

550.1 Inguinal hernia with obstruction without mention of gangrene

550.10 Inguinal hernia, with obstruction, without mention of gangrene, unilateral or unspecified (not specified as recurrent)

550.11 Inguinal hernia with obstruction, without mention of gangrene, unilateral or unspecified, recurrent

550.12 Inguinal hernia, with obstruction, without mention of gangrene, bilateral (not specified as recurrent)

550.13 Inguinal hernia, with obstruction, without mention of gangrene, bilateral, recurrent

550.9 Inguinal hernia without mention of obstruction or gangrene

550.90 Inguinal hernia, without mention of obstruction or gangrene, unilateral or unspecified (not specified as recurrent)

550.91 Inguinal hernia, without mention of obstruction or gangrene, unilateral or unspecified, recurrent

550.92 Inguinal hernia, without mention of obstruction or gangrene, bilateral (not specified as recurrent)

550.93 Inguinal hernia, without mention of obstruction or gangrene, bilateral, recurrent

**ICD-10 diagnostic codes used to identify patients with primary or recurrent inguinal hernia and, with or without, gangrene or occlusion at presentation.**

K40.0 Bilateral inguinal hernia, with obstruction, without gangrene

K40.00 Bilateral inguinal hernia, with obstruction, without gangrene not specified as recurrent

K40.01 Bilateral inguinal hernia, with obstruction, without gangrene recurrent

K40.1 Bilateral inguinal hernia, with gangrene

K40.10 Bilateral inguinal hernia, with gangrene not specified as recurrent

K40.11 Bilateral inguinal hernia, with gangrene recurrent

K40.2 Bilateral inguinal hernia, without obstruction or gangrene

K40.20 Bilateral inguinal hernia, without obstruction or gangrene not specified as recurrent

K40.21 Bilateral inguinal hernia, without obstruction or gangrene recurrent

K40.3 Unilateral inguinal hernia, with obstruction, without gangrene

K40.30 Unilateral inguinal hernia, with obstruction, without gangrene not specified as recurrent

K40.31 Unilateral inguinal hernia, with obstruction, without gangrene recurrent

K40.4 Unilateral inguinal hernia, with gangrene

K40.40 Unilateral inguinal hernia, with gangrene not specified as recurrent

K40.41 Unilateral inguinal hernia, with gangrene recurrent

K40.9 Unilateral inguinal hernia, without obstruction or gangrene

K40.90 Unilateral inguinal hernia, without obstruction or gangrene not specified as recurrent

K40.91 Unilateral inguinal hernia, without obstruction or gangrene recurrent

**ICD 9 Diagnostic codes used to identify patients with comorbidities as a secondary diagnosis**

Chronic Obstructive Pulmonary Disease, 490 to 496, 500 to 506.9

Arterial Hypertension, 401.0 to 401.9

Diabetes, 250 to 250.93

Dyslipidaemia, 272.0 to 272.9

Obesity, 278.0 to 278.02, V853, V854

Tobacco consumption/Smoking, 65.2-T65.294, F17.202 to F17.299, Z72.0

**ICD- 10 Diagnostic codes used to identify patients with comorbidities as a secondary diagnosis**

Chronic Obstructive Pulmonary Disease, J40 to J47.9

Arterial Hypertension, I10 to I16.9

Diabetes E8.10, to E13.9

Dyslipidaemia, E78 to E78.9

Obesity, E65 to E66.9

Tobacco consumption/Smoking, F17.200 to F17.299, Z72.0, and Z87.891

**ICD- 9** **Diagnostic codes used to identify patients with postoperative complications as a secondary diagnosis.**

Wound infection, 998.5 to 998.59;

Haemorrhage and/or hematoma, 998.1 to 998.12

Acute urinary retention, 788.2 to 788.29

**ICD- 10** **Diagnostic codes used to identify patients with postoperative complications as a secondary diagnosis.**

Wound infection, L01.0 to L08.9

Haemorrhage and/or hematoma, L76.02 to L76.85

Acute urinary retention, R33.0 to R33.9.
